# Impact of Premorbid Function and Comorbidities in Geriatric Traumatic Brain Injury Patients: A Systematic Review

**DOI:** 10.7759/cureus.116746

**Published:** 2026-09-22

**Authors:** Olurotimi J Badero, Sandra Bakare, Mohammad Yousaf Moosa, Opeyemi Taiwo, Saheed Hammed, Pierce Ngo, Chukwumbana Faith Okirie, Albert Abiodun Ogunlade

**Affiliations:** 1 Department of Medicine, College of Medicine, University of Lagos, Lagos, NGA; 2 Department of Interventional Cardiology, Iwosan Lagoon Hospital, Lagos, NGA; 3 Division of Cardionephrology, Cardiac Renal and Vascular Associates, Jackson, USA; 4 Department of General Research, Research Junction Institute, Columbus, USA; 5 Department of General Medicine, Atılım University, Ankara, TUR; 6 Department of Primary Care, Olabisi Onabanjo University, Ago-Iwoye, NGA; 7 Department of Education, University of Cincinnati, Cincinnati, USA; 8 Department of Medicine, Midwestern University, Glendale, USA; 9 Department of Medicine, Near East University, Nicosia, CYP; 10 Department of Physiology, Faculty of Basic Medical Sciences, Olabisi Onabanjo University, Sagamu, NGA

**Keywords:** anticoagulant use, frail, morbidity index, post-traumatic brain injury, renal health

## Abstract

Population aging is increasing geriatric traumatic brain injury (TBI), with outcomes depending on premorbid health beyond chronological age. This systematic review examined how premorbid functional status, frailty, and comorbidities influence mortality, functional recovery, and other outcomes in adults aged ≥60 years with TBI. Following the PRISMA 2020 statement, PubMed, Scopus, Web of Science, and Google Scholar were searched (2016-2026). Twenty observational studies (n = 128,793) were included. Narrative synthesis was performed; risk of bias was assessed with Joanna Briggs Institute tools. Frailty (Clinical Frailty Scale, Fatigue, Resistance, Ambulation, Illness, and Loss of weight (FRAIL) Scale, and Hospital Frailty Risk Score) and premorbid ADL dependence consistently predicted higher mortality, unfavorable Glasgow Outcome Scale-Extended scores, prolonged hospitalization, and adverse discharge disposition. Comorbidities (diabetes, chronic kidney disease, and cardiovascular disease) independently worsened neurological recovery and increased complications. Frailty measures provided prognostic value superior to age alone after adjustment for injury severity. In conclusion, premorbid functional status, frailty, and comorbidities are key determinants of outcome in geriatric TBI. Routine frailty and comorbidity assessment should be integrated into clinical evaluation and prognostic models to improve risk stratification and guide individualized management.

## Introduction and background

Population aging is increasing the number of older adults at risk for traumatic brain injury (TBI), with global estimates projecting continued growth in the older adult population by 2050 [1]. TBI is typically defined as a disruption of normal brain function or evidence of brain damage resulting from an external mechanical force [2]. TBI severity ranges from mild injury, including concussion, to severe injury associated with prolonged impaired consciousness, coma, structural intracranial lesions, or death [3,4]. The intensity depends not only on the force and location of the injury but also on individual factors such as the patient’s underlying medical conditions [5]. The Glasgow Coma Scale (GCS) is commonly used at presentation to classify injury severity, although prognosis also depends on neuroimaging findings, pupil reactivity, systemic injury, and patient-level factors.

TBI is expected to remain a leading contributor to injury-related death and disability, with falls and road traffic incidents representing major mechanisms of injury [6]. The burden of geriatric TBI is substantial because older adults experience high rates of TBI-related hospitalization, mortality, delayed recovery, and long-term functional decline compared with younger adults [7,8]. However, age alone does not fully explain prognosis after TBI. Older adults differ from younger individuals who sustain TBI because they often have age-related physiological changes and existing health conditions that can affect both the injury and recovery. Reduced physiological reserve, multimorbidity, frailty, cognitive impairment, and baseline functional limitations may reduce their ability to cope with neurological injury and can make recovery more difficult. In addition, the use of multiple medications, including anticoagulants and antiplatelet agents, is more common in older adults and may increase the risk of intracranial bleeding [9]. As a result, outcomes after TBI in older adults are influenced not only by the injury itself but also by pre-injury health status.

Frailty is increasingly recognized as a key factor influencing the risk of poor outcomes in older adults with TBI. Frailty reflects diminished physiological reserve and heightened vulnerability to stress and often overlaps with cognitive decline, reduced mobility, and dependence in activities of daily living (ADL) [10]. In patients over 65 years of age with moderate-to-severe TBI, a Clinical Frailty Scale (CFS) score of ≥4 was strongly linked with six-month mortality and unfavorable outcomes [11]. Moreover, research using the Fatigue, Resistance, Ambulation, Illness, and Loss of weight (FRAIL) Scale found that higher frailty levels predicted in-hospital death, highlighting the need for more attentive care in these patients [12]. Similarly, another study implementing the Hospital Frailty Risk Score (HFRS) showed that greater frailty was associated with longer hospital stay and poorer functional status at discharge [6].

Premorbid functional decline is defined as difficulty in performing ADL. Premorbid functional status is another important factor that may influence both the risk of sustaining TBI and the likelihood of recovery afterward [12]. In older adults who experience TBI with loss of consciousness, preexisting conditions such as impaired ADL, depression, and cerebrovascular disease have been linked to higher mortality [2]. Studies have shown that many older adults with chronic illnesses or mental health conditions report lower quality of life and worsening psychological symptoms six months after TBI [13]. Taken together, these findings suggest that frailty and premorbid health may provide prognostic information beyond chronological age alone.

Comorbidities refer to the presence of coexisting diseases and affect roughly 60-80% of older adults with TBI. These overlapping conditions shape recovery trajectories and determine the type and intensity of care each patient may require [10,14]. The most common conditions include coronary artery disease, heart failure, atrial fibrillation, dementia, and diabetes. Notably, cardiac conditions that require antithrombotic therapy influence bleeding risk and complicate acute management in TBI. Previous research suggests that diabetes and chronic kidney disease (CKD) may contribute to poorer neurological recovery and unfavorable outcomes [14,15]. Systematic reviews further indicate that comorbidity can negatively affect cognitive function and prolong rehabilitation, although the outcome varies depending on the patient’s pre-injury status [10].

Recovery after TBI in older adults can range from complete functional recovery to long-term disability or institutional care. In a long-term follow-up study of adults aged ≥60 years, von Seth et al. reported that 57% had relatively good outcomes three to 14 years after TBI, and 40% showed stable Glasgow Outcome Scale-Extended (GOSE) scores between six months and long-term follow-up [16]. Moreover, mild TBI cohorts show a high rate of independence at six months [13]. In contrast, complete recovery was uncommon when CT lesions, low socioeconomic status, and acute symptoms were present [3]. Across geriatric TBI studies, lower admission GCS, abnormal pupillary response, coagulation abnormalities, and CT findings such as midline shift, diffuse edema, and subarachnoid hemorrhage have been associated with poorer functional outcomes and higher mortality [14,16,17].

Although many studies have examined predictors of outcomes in older adults with TBI, the evidence remains limited and inconsistent. This inconsistency stems from several methodological and conceptual differences across studies. For instance, the definition of older adults varies widely, with age thresholds ranging from 60 to 90 years. In addition, study populations also differ in injury severity, with some including only moderate or severe TBI and others combining mild TBI with more severe injuries.

Prognostic models like CRASH and IMPACT are commonly used to estimate recovery after TBI. The CRASH model considers factors like age, GCS, pupillary response, and CT findings, while the IMPACT model includes additional variables such as hypotension, hypoxia, and certain laboratory tests. These tools have been valuable for predicting mortality and functional outcomes, yet they focus on injury severity and acute physiological markers. Frailty and comorbidities, which are key aspects of health in older adults, are often overlooked in CRASH and IMPACT models. This gap limits their accuracy in geriatric populations, where pre-injury health and chronic disease often shape recovery more than age alone [9]. Furthermore, frailty and comorbidity were measured using diverse instruments such as the CFS, Groningen Frailty Index, and Charlson Comorbidity Index (CCI), making comparison difficult. This lack of consistency weakens the evidence base for geriatric TBI. Because older adults with TBI may experience long-term disability, recurrent healthcare needs, and institutional care placement, geriatric TBI should be approached not only as an acute neurological event but also as a condition with chronic functional consequences. Therefore, this review aims to examine how premorbid functional status, frailty, and comorbidities influence clinical outcomes, including mortality, functional recovery, institutionalization, cognition, and quality of life, among older adults with TBI.

## Review

Methods

This systematic review was reported in accordance with the PRISMA 2020 statement. PRISMA was chosen to ensure the rigor, transparency, reproducibility, and methodological quality needed for an effective systematic review [18]. A review protocol was developed prior to study selection and registered in PROSPERO (CRD420261320032). Any deviations from the protocol were documented. Additionally, this study aimed to synthesize findings from different empirical studies on the prognostic role of premorbid functional status and comorbidities in geriatric TBI, so a systematic review was deemed appropriate for drawing reliable conclusions [19].

Study Design

The design of this study was a systematic review using narrative synthesis as the analytical strategy. Because substantial clinical and methodological heterogeneity was anticipated, findings were synthesized narratively rather than pooled statistically [20]. Eligible studies included observational study designs, including cohort, case-control, and case series with ≥10 participants. Specifically, observational studies including prospective and retrospective cohort studies, case-control studies, and cross-sectional studies were included, as these designs are the most commonly employed when investigating prognostic factors and baseline characteristics in acutely injured populations [21]. Case series with 10 or more patients were also included to capture additional evidence from settings where larger study designs may be logistically or ethically unfeasible. Randomized controlled trials were excluded unless they reported relevant observational prognostic analyses of premorbid function or comorbidity, because the review focused on naturally occurring baseline prognostic factors rather than intervention effects [22].

Search Strategy

The literature search for this review was conducted across four major databases: PubMed (MEDLINE), Scopus, Web of Science, and Google Scholar. These databases covered a broad range of medical, clinical, and rehabilitation literature and were therefore considered relevant to this review. The search was limited to studies conducted in the last 10 years (i.e., 2016-2026). The last 10 years were chosen to capture the most contemporary studies regarding geriatric TBI outcomes and older TBI management, as CT use, anticoagulant patterns, geriatric assessment, and rehabilitation pathways may differ from current practice.

MeSH terms, free-text keywords, and Boolean operators were used to construct the search strategy for this review. The search focused on three main domains involving older adults with TBI, premorbid functional status and comorbidities, and outcomes of interest. The main search terms included “traumatic brain injury”, “TBI”, “head injury”, “intracranial injury”, “geriatric”, “elderly”, “older adults”, “aged”, “premorbid function”, “functional status”, “activities of daily living”, “frailty”, “comorbidities”, “multimorbidity”, “hypertension”, “diabetes mellitus”, “cardiovascular disease”, “dementia”, “prognosis”, “mortality”, “functional outcome”, and “discharge disposition”. Backward citation searching was also conducted as a supplementary search to identify other relevant literature.

Eligibility Criteria

The Population, Intervention/Exposure, Comparator, Outcome, and Study Design (PICOS) framework was used to define the eligibility criteria for this review in order to maintain consistent and systematic application during the screening process [23]. The criteria were defined in terms of the population, exposure (premorbid functional status and comorbidities), comparators, outcomes, and study design. Eligible populations included male and female adults aged ≥60 years with a clinical diagnosis of TBI. Studies with mixed-age samples were included only if data for participants aged ≥60 years were reported separately or could be extracted. For exposure, included studies were required to assess premorbid functional status (e.g., dependence in ADL, frailty scores such as the CFS or Frailty Index, or other validated functional scales) and/or preexisting comorbid conditions (e.g., hypertension, diabetes mellitus, cardiovascular disease, dementia, or composite multimorbidity indices) as primary or secondary exposures of interest. For comparators, included studies needed to enable comparison between patients with different levels of premorbid function or different comorbidity burdens (e.g., presence versus absence of specific comorbidities, low versus high frailty scores, or independent versus dependent premorbid ADL status) and report outcomes according to premorbid functional status, frailty, or comorbidity status, either through stratified descriptive results or effect estimates.

Additionally, the primary outcome of interest of the included studies was mortality, including in-hospital mortality, 30-day mortality, and long-term mortality. Secondary outcomes included functional recovery, discharge disposition (e.g., home, rehabilitation facility, or nursing home), length of hospital stay, ICU admission, complications during hospitalization, and health-related quality of life. Lastly, the included studies had to use observational study designs (cohort, case-control, and cross-sectional studies) or case series with ≥10 patients and be published in English from January 2016 onward. Case reports, case series with fewer than 10 patients, narrative or systematic reviews, editorials, letters to the editor, conference abstracts without accessible full texts, and nonhuman studies were excluded, as shown in Table 1.

**Table 1:** Inclusion and exclusion criteria. TBI, traumatic brain injury

Domain	Inclusion criteria	Exclusion criteria
Population	Adults aged ≥60 years with clinically diagnosed TBI	Nonhuman studies; studies without separable older adult data
Exposure	Premorbid functional status, frailty, comorbidity, or multimorbidity	Studies not reporting relevant premorbid or comorbidity data
Outcomes	Mortality, functional recovery, discharge disposition, length of stay, ICU admission, complications, quality of life	Studies without relevant clinical outcomes
Study design	Cohort, case-control, cross-sectional studies, and case series with ≥10 participants	Case reports, case series with <10 participants, reviews, editorials, letters, conference abstracts without full text
Publication characteristics	English-language full-text studies published from January 2016 onward	Non-English studies; unavailable full texts

Study Selection

**Figure 1:**
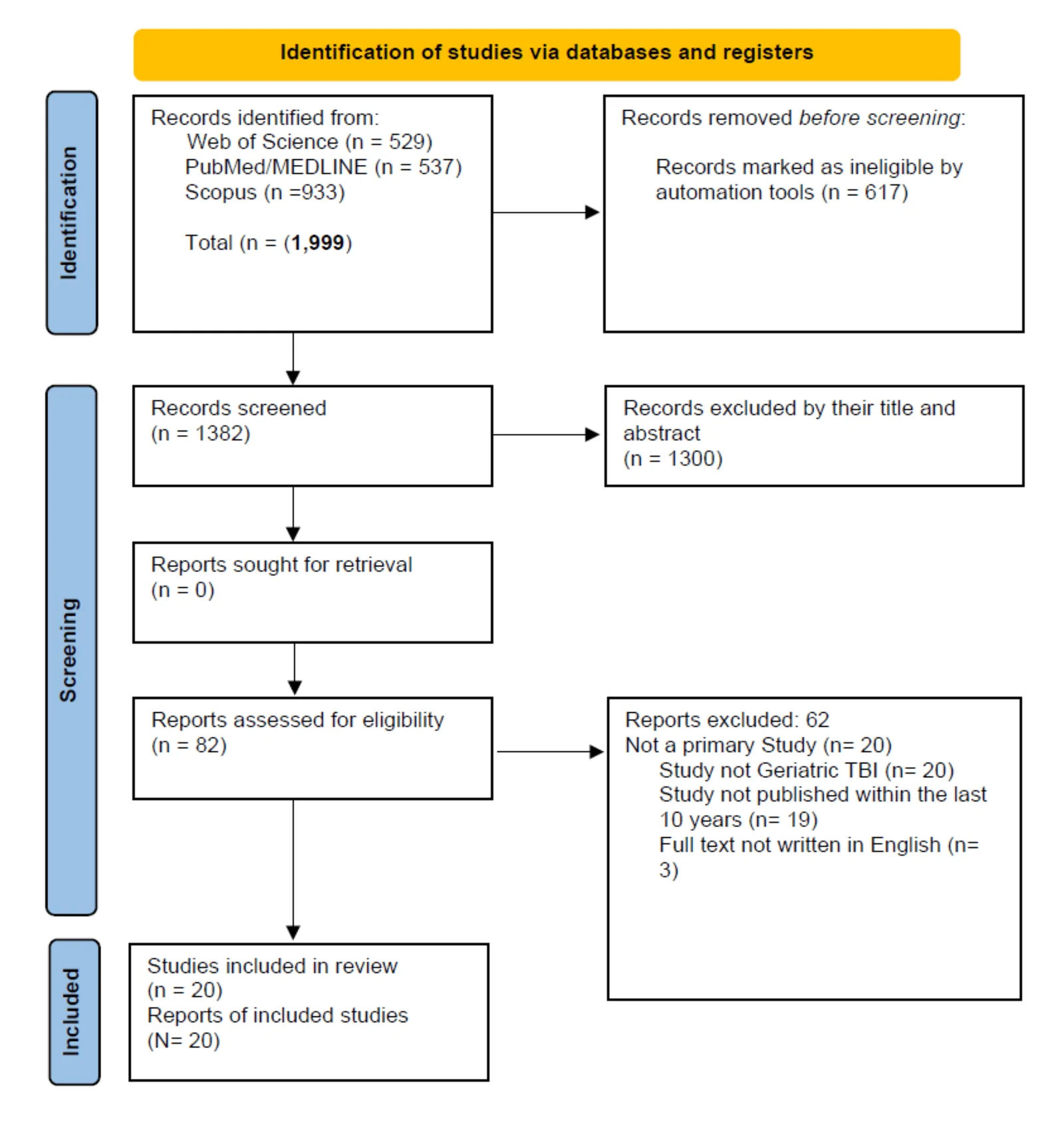
PRISMA flow diagram of included studies. [18]

The study selection process was conducted in two sequential phases, namely, title and abstract screening followed by full-text review, in accordance with PRISMA 2020 recommendations [18]. Two reviewers independently screened titles and abstracts. Full texts of potentially eligible articles were then assessed independently by the same reviewers. Disagreements were resolved through discussion or consultation with a third reviewer. Reasons for exclusion at the full-text stage were recorded. To facilitate collaborative and efficient screening, Rayyan was utilized [24]. Figure 1 presents the PRISMA 2020 flow diagram showing the study selection process.

Data Synthesis

Findings were synthesized narratively. Studies were grouped according to exposure domain: premorbid functional status, frailty, individual comorbidities, and composite comorbidity burden. Outcomes were grouped as mortality, functional recovery, discharge disposition, length of stay, ICU admission, complications, and quality of life. Adjusted estimates were prioritized over unadjusted estimates when available. The direction, magnitude, and precision of associations were summarized, and heterogeneity was explored according to TBI severity, outcome timing, study design, and risk-of-bias rating.

Data Extraction

Data extraction was performed independently by two reviewers using a standardized extraction form. Extracted data were compared, and discrepancies were resolved by consensus. Data extracted for this study included study citation details; study design and setting; sample size and patient characteristics (mean age, sex distribution, injury mechanism, and GCS at presentation); definition and measurement of premorbid functional status; specific comorbidities reported and tools used for comorbidity assessment; primary and secondary outcomes reported; follow-up duration; statistical methods used; and key findings, including measures of association (ORs, HRs, or RRs) with corresponding CIs where available. All tools were free to use.

Results

From the databases searched, 1,999 records were identified: 529 from Web of Science, 537 from PubMed/MEDLINE, and 933 from Scopus. Before screening, 617 records were excluded using automated filters that removed duplicates. Two reviewers verified these exclusions prior to title and abstract screening. The remaining 1,382 articles were screened by title and abstract. Of the 82 full-text reports assessed for eligibility, 62 were excluded: 20 were not relevant to geriatric TBI, 19 were published outside the eligible date range, 20 were not primary studies, and three were not written in English. Twenty studies were included in the final review.

The included studies were published between 2016 and 2025 and were conducted across Europe, North America, and Asia. Most included studies were retrospective cohort studies (n = 13). The remaining studies included prospective cohort studies (n = 4), mixed prospective-retrospective cohorts (n = 2), and one cross-sectional long-term follow-up cohort. The final review included 20 studies comprising 128,793 participants. All focused on patients aged 60 years and above with TBI or related intracranial pathologies. Two studies focused specifically on chronic subdural hematoma (CSDH). These were retained because they examined older adults with trauma-associated intracranial pathology and directly evaluated premorbid frailty or comorbidity as predictors of mortality and functional outcomes. Characteristics and synthesis of the included studies are presented in Tables 2, 3.

**Table 2:** Characteristics of included studies. ADL, activities of daily living; aCCI, age-adjusted Charlson Comorbidity Index; AIS, Abbreviated Injury Scale; APOE, apolipoprotein E; ASA, American Society of Anesthesiologists; AUC, area under the curve; BI, Barthel Index; BIMS, Brief Interview for Mental Status; CCI, Charlson Comorbidity Index; CFS, Clinical Frailty Scale; CKD, chronic kidney disease; CSDH, chronic subdural hematoma; DNR, do not resuscitate; EDH, epidural hematoma; ESRD, end-stage renal disease; FIM, Functional Independence Measure; GCS, Glasgow Coma Scale; GFI, Groningen Frailty Indicator; GNRI, Geriatric Nutritional Risk Index; GOSE, Glasgow Outcome Scale-Extended; GOS, Glasgow Outcome Scale; HADS, Hospital Anxiety and Depression Scale; HDaH, Healthy Days at Home; HFRS, Hospital Frailty Risk Score; ICD-10, International Classification of Diseases, Tenth Revision; IDI, integrated discrimination improvement; IRF, inpatient rehabilitation facility; IRF-PAI, Inpatient Rehabilitation Facility-Patient Assessment Instrument; ISS, Injury Severity Score; JCS, Japan Coma Scale; LOS, length of stay; LOC, loss of consciousness; MCID, minimally clinically important difference; MDC, minimal detectable change; MPAI-4, Mayo-Portland Adaptability Inventory-4; mSHE, modified Subdural Hematoma in the Elderly score; NRI, net reclassification improvement; NTBI, non-traumatic brain injury; PTA, post-traumatic amnesia; PTSD, posttraumatic stress disorder; QoL, quality of life; ROC, receiver operating characteristic; RPQ, Rivermead Post-Concussion Symptoms Questionnaire; SAH, subarachnoid hemorrhage; SDH, subdural hematoma; SF-12, 12-Item Short Form Health Survey; SHE, Subdural Hematoma in the Elderly score; SNF, skilled nursing facility; TBI, traumatic brain injury; TRISS, Trauma and Injury Severity Score; TUG, Timed Up and Go

Study	Study design	Sample size and population	Age	Premorbid function assessed and comorbidities considered	TBI severity	Outcomes measured	Primary/key findings	Secondary findings
Dams-O’Connor et al. (2016) [2], USA	Prospective community-based cohort study (adult changes in thought study)	N = 3,363 adults ≥65 years with no prior TBI with LOC, followed for a mean of 7.5 years (up to 18 years)	Mean age ~74.6 years	Premorbid health factors assessed: depression (CES-D score), ADL impairment, cerebrovascular disease, chronic disease comorbidity index, heart disease, sedentary lifestyle, cognitive function, alcohol problems, APOE ε4 genotype	Incident TBI with LOC recorded during follow-up	Incident TBI risk and post-TBI mortality analyzed using Weibull survival models	76 participants (2.3%) developed incident TBI with LOC. Independent predictors included depression (HR 1.23 per 4 CES-D points; 95% CI 1.02-1.49; p = .03) and ADL impairment (HR 2.37; 95% CI 1.24-4.53; p = .009). In time-varying models, cerebrovascular disease predicted TBI risk (HR 2.28; 95% CI 1.37-3.78; p < .001).	Among individuals who developed TBI, ADL impairment (HR 3.71; 95% CI 1.53-8.95; p = .004) and cerebrovascular disease (HR 2.40; 95% CI 1.21-4.75; p = .01) predicted higher post-TBI mortality. These results suggest preexisting health, mood, and functional decline precede and predict TBI in older adults.
Horvat et al. (2025) [3], Serbia	Combined prospective-retrospective cohort study conducted at a tertiary neurosurgical center (June 2022-May 2023)	N = 93 older adults presenting with mild TBI; the majority sustained injuries from falls.	Ages ranged from 65 to 89 years, with a mean age of 72.1 ± 6.8 years.	Education level, marital status, living situation, social support; comorbidities included hypertension, diabetes, cardiac disease, psychiatric history, alcohol use, and anticoagulant therapy	Mild TBI (GCS 13-15); classified as uncomplicated vs CT-positive intracranial lesion	GOSE, SF-12, RPQ, PHQ-9, PCL-5, TUG, 10-m walk (six-month follow-up)	94.9% achieved functional independence, but only 43% achieved full recovery. CT-positive lesions were associated with worse mobility (TUG 17.6 vs 16.3 s; p = 0.012). Severe acute headache predicted poorer physical QoL (p = 0.010) and lower GOSE. Lower educational level was associated with poorer recovery (p = 0.025). Depression/PTSD was not associated with CT lesions (p = 0.60).	Education level independently influenced global recovery measured by GOSE (p = 0.010). Increasing age was associated with poorer GOSE recovery (p = 0.026).
Zhu et al. (2023) [4], China	Retrospective cohort study	N = 228 elderly patients with mild TBI treated at Beijing Tiantan Hospital (2013-2018)	Mean age ~74-76 years; all ≥65 years	Premorbid factors assessed included hypertension, diabetes, heart disease, stroke history, cancer, anemia, anticoagulant/antiplatelet use, comorbidity burden (age-adjusted CCI, comorbidity-polypharmacy score). Nutritional status was assessed using the GNRI.	Mild TBI (GCS 13-15); LOC ≤30 min and post-traumatic amnesia <24 h	Primary: functional recovery at six months using GOSE (complete recovery, GOSE = 8, vs incomplete recovery, GOSE ≤7). Secondary: predictors of incomplete recovery	Low GNRI (malnutrition) was strongly associated with incomplete recovery. Each increase in GNRI reduced the risk of incomplete recovery (adjusted OR 0.770, 95% CI 0.709-0.837, p < 0.001). Patients with high GNRI ≥97.85 had a significantly lower risk of incomplete recovery (OR 0.047, 95% CI 0.020-0.109, p < 0.001). GNRI predicted recovery with an AUC of 0.860 (95% CI 0.806-0.911, p < 0.001).	Incomplete recovery was associated with older age (p = 0.001), anemia (p < 0.001), higher comorbidity burden (aCCI, p = 0.008), lower albumin and hemoglobin (p < 0.001), and acute subdural hematoma (p = 0.004). Adding GNRI to conventional models significantly improved prediction of poor recovery (NRI 126.17%, p < 0.001; IDI 25.0%, p < 0.001).
Yamamoto et al. (2022) [6], Japan	Historical retrospective cohort (nationwide database study)	N = 18,065 hospitalized TBI patients from the JMDC database (2014-2020)	Mean 71.8 years; majority ≥75 years in higher frailty groups	Premorbid status assessed using HFRS derived from ICD-10 comorbidities (e.g., dementia, delirium, neuropathy, fractures, urinary disorders, hypotension). Frailty groups: low (<5), intermediate (5-15), high (>15)	TBI types included diffuse TBI, focal TBI, epidural hemorrhage, traumatic subdural hemorrhage, and traumatic subarachnoid hemorrhage. Severity partly assessed using JCS and baseline Barthel Index	LOS, Barthel Index ≥95 at discharge, Barthel Index gain, in-hospital mortality	Higher frailty was associated with longer hospital stay (intermediate: coefficient 1.952, 95% CI 1.117-2.786; high: 5.770, 95% CI 3.160-8.379; p < 0.001). Frailty was associated with lower functional recovery: BI ≥95 at discharge (intermediate: OR 0.645, 95% CI 0.595-0.699; high: OR 0.221, 95% CI 0.157-0.311; p < 0.001). Frailty was associated with lower BI gain (intermediate: -4.868, 95% CI -5.599 to -3.773; high: -19.596, 95% CI -22.242 to -16.714; p < 0.001). Frailty was not significantly associated with in-hospital mortality (intermediate OR 0.901, p = 0.690; high OR 0.707, p = 0.245).	Intermediate- and high-frailty groups had longer LOS, lower ADL independence, and lower functional improvement. Frailty prevalence in the TBI cohort was ~44%. HFRS may be better for predicting functional outcomes rather than mortality in TBI patients.
Zacchetti et al. (2023) [8], Italy	Prospective observational single-center cohort study	N = 60 patients ≥65 years admitted to a neurosurgical ICU with TBI	Median 76 years (IQR 70-80); 65% male	Premorbid frailty assessed using CFS; comorbidities included neurodegenerative disease (30%), cardiopulmonary disease (50%), antiplatelet therapy (38%), anticoagulant use (20%)	Moderate-severe TBI (GCS ≤13); median admission GCS 8 (IQR 6-11)	Primary: functional outcome at six months using GOSE. Secondary: mortality, clinical predictors	Frailty (CFS ≥4) was strongly associated with poor outcomes. Six-month mortality: 87% in frail vs 30% in non-frail (OR 15.7, 95% CI 3.9-55.2, p < 0.001). Unfavorable functional outcome: 92% vs 51% (OR 9.9, 95% CI 2.1-46.3, p = 0.002). In multivariate analysis, frailty remained independently associated with poor outcome (OR 7.84, 95% CI 1.40-43.97, p = 0.019).	Univariate predictors of poor outcome included male sex (p = 0.029), neurodegenerative disease (p = 0.027), lower GCS (p = 0.008), pupillary abnormalities (p = 0.031), basal cistern compression (p = 0.017), and traumatic SAH (p = 0.011). However, in multivariate analysis, only frailty (CFS ≥4) and traumatic SAH (OR 5.47, 95% CI 1.13-26.58, p = 0.035) remained significant predictors of unfavorable outcome.
Hernández-Durán et al. (2022) [9], Germany	Retrospective cohort study	N = 168 elderly patients with CSDH after minor or no trauma	Median age 79 years (IQR 10.75)	Frailty assessed using the CFS and incorporated into a modified Subdural Hematoma in the Elderly score (mSHE); anticoagulant/antiplatelet use recorded (61%)	CSDH in elderly patients	Primary: 30-day mortality. Secondary: functional outcome at 30 days using GOS	Mortality 4% (n = 7). The original SHE score failed to predict mortality (AUC 0.564, p = 0.565). The mSHE predicted mortality significantly (AUC 0.749, p = 0.026).	mSHE also predicted functional outcome (AUC 0.862, p < 0.001). A threshold mSHE ≥3 predicted mortality (sensitivity 50%, specificity 75%) and poor outcome (sensitivity 88%, specificity 64%). Frailty improved prognostic accuracy beyond traditional predictors.
Abdulle et al. (2018) [10], Netherlands	Prospective observational cohort study (UPFRONT cohort) conducted in three Dutch Level 1 trauma centers	N = 161 elderly patients ≥60 years with mild TBI	Mean age 70.8 ± 6.3 years	Pre-injury health status included physical impairments in 37% of patients (51% frail vs 29% non-frail, p < 0.01) and preexisting mental health problems in 6%. Overall comorbidity burden was present in 65% of the cohort. Frailty was assessed using the GFI, with 37% classified as frail, reflecting multidimensional deficits across physical, cognitive, social, and psychological domains.	Mild TBI defined as GCS 13-15, LOC ≤30 min and/or PTA <24 h. 72% had GCS 15 on admission; mean ISS 8.2 ± 5.9	Early outcomes: post-traumatic complaints and emotional distress (HADS) at two weeks. Long-term outcomes: Extended GOSE and frailty (GFI) at 1-3 years post-injury	Frailty independently predicted unfavorable long-term outcome (OR 2.1, 95% CI 1.59-2.77, p < 0.001). Presence of early post-traumatic complaints also predicted poor outcome (OR 1.13, 95% CI 1.01-1.27, p = 0.04).	54% completely recovered, 14% mild disability, 22% moderate disability, 11% severe disability. Frail patients had significantly worse recovery (24% full recovery vs 72% in non-frail, p < 0.01). Early depression was more common in frail patients (26% vs 7%, p = 0.001). Frail patients had lower life satisfaction scores (6.7 vs 7.9, p < 0.01) and more functional limitations at follow-up.
Kumar et al. (2025) [15], India	Retrospective cohort	N = 654 geriatric patients (≥60 years) with TBI treated at a tertiary neurosurgical center (2016-2020)	Mean age 66.16 ± 6.55 years; majority 60-70 years (82.6%)	Comorbidities assessed included diabetes mellitus, hypertension, and other chronic illnesses. LOC and altered sensorium at presentation were also recorded.	Based on GCS: 41% severe TBI (GCS <9), remainder moderate or mild	GOS at discharge and six-month follow-up, hospital mortality, clinical and radiological characteristics	Hospital mortality 25.2%. 60.7% of discharged patients had favorable outcomes (GOS 4-5). GCS at admission and best motor response strongly predicted favorable outcomes (p < 0.001). History of LOC and altered sensorium was associated with unfavorable outcomes (p < 0.001). Diabetes mellitus predicted poorer outcomes at six months (p < 0.001), while other comorbidities were not significantly associated.	The most common CT abnormality was subdural hematoma. Falls and road traffic accidents were the most common mechanisms of injury. Patients presenting with severe GCS scores had significantly higher mortality (p < 0.001).
von Seth et al. (2026) [16], Sweden	Cross-sectional cohort (long-term follow-up of TBI survivors in the Uppsala Long-term Outcome in Older Adults with TBI Study)	N = 79 older adults surviving TBI; majority male (65%). Falls were the most common mechanism (~68%).	Mean age 76 ± 5 years; range 64-91 years; mean time since injury 7 ± 3 years	Sociodemographic factors included marital status, vocational status, need for assistance, rehabilitation status, and use of mobility devices. Comorbidities included cardiovascular disease (43%), anticoagulation therapy, prior brain disease, dysphagia, and sensory impairments.	Mixed severity TBI: mild 56%, moderate 37%, severe 8% based on consciousness level (Reaction Level Scale classification)	GOSE, FIM, MPAI-4, and sociodemographic and clinical factors at long-term follow-up	Injury severity significantly correlated with functional outcomes: GOSE (p = 0.026), FIM cognition (p < 0.001), FIM motor (p = 0.033), and MPAI-4 ability (p = 0.038). The majority had relatively good functional outcomes on MPAI-4, and 40% showed no change in GOSE between six-month and long-term follow-up. Injury severity was the primary determinant of long-term outcome.	Older age was associated with worse participation and adjustment scores on the MPAI-4. Individuals with greater premorbid disability had significantly poorer social participation outcomes. Cognitive and emotional subscales of MPAI-4 showed greater impairment in older adults compared with younger cohorts.
Sharma et al. (2021) [17], India	Mixed retrospective and prospective analytical cohort study	N = 160 patients ≥65 years admitted with TBI at a tertiary hospital (2016-2018)	Mean age 71.08 years; 75.6% male	Comorbidities assessed: diabetes (25.6%), coronary artery disease/hypertension (40.6%), chronic liver disease (3.1%), substance use, and other chronic illnesses. Laboratory factors included hemoglobin, creatinine, and random blood sugar	Mixed TBI severity with CT-confirmed lesions (e.g., subdural hematoma 60.6%, contusions 26.9%, intracerebral hematoma 10.6%). Admission GCS differed between outcome groups	Primary: mortality and discharge outcome (GOS). Secondary: predictors of mortality, including clinical and laboratory factors	Lower GCS on arrival was strongly associated with mortality (p = 0.000). Non-reactive pupils significantly predicted poor outcome. Low hemoglobin, high random blood sugar, and elevated creatinine were significantly associated with mortality. Patients who died or were discharged against medical advice had significantly worse neurological status.	Radiologic predictors of poor outcome included skull fracture, cerebral infarct, diffuse brain edema, subarachnoid hemorrhage, and midline shift. Ventilatory support was strongly associated with mortality (p = 0.000). Anticoagulant use and comorbidities were not associated with increased mortality. Overall mortality in the cohort was 3.8%.
Evans et al. (2022) [25], USA	Retrospective cohort study using linked Medicare administrative data + National Trauma Data Bank	Mean n = 1,178 older adults ≥66 years hospitalized for TBI and discharged to IRFs (2011-2015)	Mean age 79.7 years (95% CI 78.7-80.6)	Baseline functional status assessed using FIM motor and cognitive scores at rehabilitation admission, reflecting pre-injury functional reserve. Comorbidity burden measured using the CCI (mean 1.6, 95% CI 1.4-1.7), with pre-injury dementia present in 21.7% of patients.	GCS severity: mild 83.4%, moderate 3.9%, severe 7.4%; AIS head injury severity 1-5; ICU admission 80.3%	Functional motor improvement measured using FIM-Motor scores; MDC (≥9 points); MCID (≥17 points); FIM-Motor discharge score	84.1% achieved MDC and 68.6% achieved MCID in motor function during inpatient rehabilitation.	Higher CCI reduced odds of achieving MCID (OR 0.88, 95% CI 0.79-0.98). Higher cognitive function increased odds of MCID (OR 1.85, 95% CI 1.10-3.12). Age was not significantly associated with achieving MCID (p = 0.41), but age ≥85 years predicted lower discharge motor function (β -3.87, 95% CI -7.29 to -0.45).
Kiwanuka et al. (2025) [26], Sweden	Retrospective single-center cohort study (Swedish Trauma Registry)	N = 823 trauma patients: 244 TBI and 579 non-TBI controls	TBI median 67 years (IQR 51-78); NTBI 57 years (37-76)	ASA score used to assess pre-injury comorbidity burden; anticoagulant and antiplatelet use recorded	Complicated mild TBI (GCS 13-15) with intracranial lesions; 97.5% had AIS head ≥2	Primary: 90-day mortality. Secondary: hospital stay, GOS at discharge, TRISS survival probability	Age and ASA ≥3 independently predicted 90-day mortality in TBI: age OR 1.04 (95% CI 1.00-1.09, p = 0.034); ASA ≥3 OR 3.44 (95% CI 1.10-13.41, p = 0.048). Overall 90-day mortality = 8.2% in TBI vs 5.4% in NTBI.	Univariable predictors in TBI included age (OR 1.06, p = 0.001), ASA ≥3 (OR 7.06, p < 0.001), AIS head ≥3 (OR 3.11, p = 0.048), and anticoagulants (OR 4.74, p = 0.009). In NTBI patients, only age remained significant in multivariable analysis. Low-energy falls were the most common mechanism (59% of TBI).
Blaauw et al. (2022) [27], Netherlands	Retrospective multicenter cohort study with matched population controls	N = 1,307 patients with CSDH treated at 3 Dutch neurosurgical centers (2005-2019); compared with 5,228 matched controls	Mean 73.7 ± 11.4 years; 73% male	Frailty assessed using 6 indicators: cognitive problems, frequent falling, inability to live independently, inability to perform daily self-care, benzodiazepine/psychotropic use, and number of medications. Comorbidities assessed using CCI	CSDH severity measured using GCS, Markwalder Grading Scale, and imaging parameters (midline shift). 78.7% underwent surgery	Primary: mortality and survival. Secondary: causes of death and association of frailty indicators with survival	CSDH patients had higher mortality than matched population controls (HR 1.34, 95% CI 1.2-1.5, p < 0.0001). Frailty indicators were independently associated with mortality: frequent falling (HR 1.3, 95% CI 1.0-1.7), inability to live independently (HR 1.4, 95% CI 1.1-1.8), inability to perform daily self-care (HR 1.5, 95% CI 1.1-1.9), number of medications used (HR 1.0, 95% CI 1.0-1.1). Older age (HR 1.05 per year) and higher CCI scores (HR 1.3 per point) also predicted mortality. Surgical treatment was associated with lower mortality (HR 0.48, 95% CI 0.32-0.72).	Median follow-up ~56 months. 40% mortality among CSDH patients during follow-up. Major causes of death were cardiovascular disease (37% vs 30% in controls, p < 0.001) and accidents/falls (7.2% vs 3.7%, p = 0.001). Frailty indicators such as functional dependence and fall risk strongly predicted mortality.
Mo et al. (2023) [28], China	Retrospective case-control study	N = 38 older adults with TBI (11 with CKD, 27 matched controls without CKD)	Mean 70.5 ± 4.5 years; 81.6% male	Comorbidities assessed included CKD, hypertension, diabetes, coronary heart disease, and stroke; laboratory markers included hemoglobin, albumin, serum urea, and creatinine	TBI with mixed intracranial lesions (SDH 55.3%, cerebral contusion 65.8%, EDH 13.2%); admission GCS 11.6 ± 2.7	Primary: discharge GCS score, infection during hospitalization, ICU stay, hospital stay, costs. Secondary: change in GCS (discharge vs admission) indicating unfavorable outcome	CKD patients had significantly worse neurological outcomes: discharge GCS 7.1 ± 5.9 vs 13.1 ± 2.6 in non-CKD (p = 0.007). Hospital-acquired infection was higher in the CKD group (54.5% vs 7.4%, p = 0.004). Logistic regression identified predictors of poor prognosis: older age (OR 1.30, 95% CI 1.06-1.60, p = 0.013), lower admission GCS (OR 0.56, 95% CI 0.37-0.85, p = 0.006), higher serum urea (OR 1.47, p = 0.006), and higher creatinine (OR 1.01, p = 0.017).	CKD patients showed higher prevalence of hypertension (90.9%), diabetes (45.5%), and coronary heart disease (63.6%) compared with controls (p < 0.05). ROC analysis showed urea AUC 0.877 and creatinine AUC 0.828 for predicting unfavorable TBI outcomes. CKD patients also had longer ICU stays and higher costs, though not statistically significant.
Su et al. (2021) [29], Taiwan	Retrospective cohort study	N = 581 elderly patients with TBI treated at a trauma center (2009-2019)	All ≥65 years; elderly cohort with mean age ~mid-70s	Premorbid status assessed using GNRI based on serum albumin, weight, and height. Comorbidities included hypertension, coronary artery disease, diabetes, congestive heart failure, ESRD, and other chronic illnesses	Moderate-severe TBI defined by AIS head ≥3; injury severity also measured with GCS and ISS	Primary: in-hospital mortality. Secondary: association between GNRI risk categories and mortality	GNRI was an independent predictor of mortality. Multivariate logistic regression showed GNRI, ESRD, and ISS were independently associated with mortality (p < 0.001). Patients with higher nutritional risk had significantly higher mortality compared with those with normal nutritional status.	Patients in the lowest GNRI categories (highest nutritional risk) had significantly greater mortality than those with GNRI >98. The mortality group had lower albumin levels, higher ISSs, and more comorbidities compared with survivors. GNRI demonstrated strong prognostic value for mortality prediction in elderly patients with moderate-severe TBI.
Kumar et al. (2024) [30], USA	Retrospective cohort study using linked Medicare claims + National Trauma Databank data	Mean N = 631 community-dwelling Medicare beneficiaries ≥66 years hospitalized for TBI (2012-2014) and admitted to IRFs within 72 hours of hospital discharge	Median age 79.6 years (95% CI 78.5-80.7). Age distribution: 66-74 years 28.2%, 75-84 years 44.0%, 85-99 years 27.8%	Functional status measured using FIM self-care, mobility, and cognitive scores at inpatient rehabilitation discharge. Comorbidity burden assessed using the CCI (median 0.6, 95% CI 0.5-0.8); acute hospital complications included deep vein thrombosis (2.4%), urinary tract infection (5.8%), and pneumonia (4.9%).	GCS: mild 88.4%, moderate 4.1%, severe 7.4%. AIS head distribution: 1-5 (most commonly AIS 4 = 47.2%). Mechanism: falls 75%	Primary outcome: HDaH in the year after inpatient rehabilitation discharge (days alive without inpatient hospital, nursing home, or home health utilization). Secondary measures: rehabilitation functional outcomes (FIM self-care, mobility, cognition), discharge destination, health-care utilization patterns	HDaH declined significantly after TBI. Patients spent 93.2% of the year before TBI at home without care vs 65.3% in the year after IRF discharge. Among survivors only, HDaH was 73.6%. Greater HDaH was significantly associated with higher FIM mobility score at rehabilitation discharge (β = 0.03; 95% CI 0.002-0.06; p = 0.034).	CCI showed a borderline association with lower HDaH (β = -0.06; 95% CI -0.13-0.001; p = 0.057). Other predictors, including age, sex, race, injury severity (AIS), mechanism of injury, neurosurgery, hospital complications, ICU stay, and rehabilitation LOS, were not statistically significant. Discharge disposition strongly influenced outcomes: HDaH 77.7% when discharged home, 47.0% when discharged to a nursing home, and 46.1% when discharged to an acute hospital.
Sastry et al. (2022) [31], USA	Retrospective single-center cohort study	N = 100 patients ≥70 years admitted for non-operative TBI to a Level I trauma center	Mean 82.2 years (low frailty 80.1 vs high frailty 83.4; p = 0.032)	Premorbid function assessed using the FRAIL score (0-1 low frailty; 2-5 high frailty). Medication/comorbidity factors included antiplatelet or anticoagulant use (79%).	Majority mild TBI: 90% GCS 14-15, 6% moderate (9-13), 4% severe (3-8). Common injuries: acute subdural hematoma 63%, traumatic SAH 33%, intracerebral hemorrhage 20%.	Primary: unfavorable discharge disposition (SNF, hospice, or death). Secondary: major inpatient complications, LOS, 30-day readmission, neurosurgical follow-up	44% had unfavorable discharge overall. High-frailty patients had significantly higher unfavorable discharge (53.8% vs 25.7%, p = 0.007). In multivariate analysis, frailty independently predicted unfavorable discharge (aOR 2.63, 95% CI 1.02-7.14, p = 0.005), and increasing age also predicted worse discharge outcomes (aOR 1.11, 95% CI 1.04-1.20, p = 0.004).	Mean hospital LOS 3.78 days. 7% developed major inpatient complications. Thirty-day readmission was 13%. Frailty was not associated with LOS, complications, or readmission on univariate analysis. High-frailty patients were more frequently transferred to another hospital service (p = 0.042).
Deutsch et al. (2024) [32], USA	Retrospective descriptive study using national administrative database (IRF-PAI)	N = 99,804 Medicare beneficiaries with TBI treated in U.S. IRFs (2013-2018)	All ≥65 years; large national elderly cohort	Prior to injury, ~85% of patients were independent in self-care and ~89% independent in indoor mobility. A large proportion had a history of falls (>75%), and approximately one-third used a walker prior to injury. Comorbidity burden was captured using the IRF tiered comorbidity classification system, with notable prevalence of tier-3 comorbidities among the rehabilitation cohort.	Severity not stratified using GCS categories; functional status and impairment severity assessed using FIM and case-mix classification	Functional outcomes (self-care and mobility), communication and cognitive status, LOS, discharge destination, complications during rehabilitation	Number of older TBI patients treated in inpatient rehabilitation increased from 14,657 in 2013 to 18,791 in 2018 (28.2% increase). Functional outcomes improved during rehabilitation, with self-care FIM change scores increasing from 11.4 to 12.4 and mobility change scores from 10.7 to 11.5 across the study period. Proportion discharged to the community increased from 67.8% to 71.6%.	Cognitive impairment was common: ~29-30% moderate impairment and ~13-14% severe impairment based on BIMS. Falls during rehabilitation occurred in ~7% of patients, with major injury in ~0.2-0.3%. Around 27% required tube feeding at admission, and ~7% had pressure ulcers on admission. Median therapy exposure during rehabilitation was ~375 minutes/week for physical and occupational therapy and ~216-225 minutes/week for speech therapy.
Gavrila Laic et al. (2023) [33], Belgium	Retrospective cohort (20-year single-center study)	N = 1,480 patients ≥65 years with TBI treated at a tertiary neurosurgery center (1999-2019)	Median 78 years (IQR 12); 68.5% ≥75 years	Premorbid factors assessed included ADL dependency (8%), nursing home residence (5.8%), cardiovascular disease (52.8%), anticoagulant use (35.5%), polypharmacy (37.8%), musculoskeletal problems, dementia, Parkinson’s disease, and alcohol abuse	All severities included; median GCS 15. Mild TBI most common. Injury types: subdural hematoma 28.4%, skull fracture 24.5%, SAH 22.9%, brain contusion 18.2%	Clinical management (ICU admission, neurosurgery), 30-day mortality, ADL dependency, nursing home admission, long-term neurological diagnoses	Falls were the most common mechanism (79.7%). 11% died within 30 days. ICU admission 27%, neurosurgery 16.4%. Dependency after TBI occurred in 24.4%, nursing home admission 17.5%. Older age was associated with higher mortality and DNR orders (p < 0.01). Higher age groups also had more comorbidities (p < 0.01).	Falls occurred mostly at home; cardiovascular disease and polypharmacy were common in elderly TBI patients. Over the 20-year period, there was increasing patient age and comorbidity burden, reduced ICU admissions and neurosurgical interventions, and decreasing mortality rates. Mild TBI still had significant consequences, with 6.9% 30-day mortality and substantial hospitalization rates.
Visen et al. (2024) [34], India	Prospective cohort study at a tertiary neurosurgical center	N = 220 geriatric patients (≥60 years) with TBI admitted to hospital	Mean 69.17 ± 7.47 years (range 60-88); 70.9% male	Comorbidities included hypertension (48.9%), diabetes mellitus (34.7%), alcohol dependence (14.6%), antiplatelet use (23.9%), anticoagulant use (1.8%), and coagulation abnormalities (27.4%)	Based on GCS: 56.2% mild, 21.9% moderate, 21.9% severe TBI. CT severity assessed with Rotterdam score	GOS at one-month follow-up, mortality, complications, and clinical predictors	54.1% had favorable outcome (GOS 4-5) and 45.9% unfavorable (GOS 1-3). Mortality 30.9% at one month. Poor outcomes were strongly associated with TBI severity (p < 0.001) and higher Rotterdam CT score (p < 0.001). Coagulation abnormalities independently predicted poor outcome (multivariable OR 2.92, 95% CI 1.30-6.57, p = 0.010). Lower admission GCS strongly predicted worse outcomes (OR 0.63, 95% CI 0.54-0.73, p < 0.001). Alcohol dependence was also associated with poorer outcome (p = 0.015).	Individuals with prior TBI showed higher thrombin generation compared with controls. TBI history was associated with alterations in clot formation parameters even after adjusting for age, sex, and medication use. Coagulation abnormalities remained associated with vascular comorbidities such as hypertension and cardiovascular disease.

**Table 3:** Synthesis of factors and outcomes in older adults with TBI. OR >1 indicates higher odds of the reported adverse outcome, whereas OR <1 indicates lower odds. Statistical significance was defined as p < 0.05. Effect estimates are reported as presented in the individual studies. ADL, activities of daily living; aOR, adjusted OR; ASA, American Society of Anesthesiologists; β, regression coefficient; CFS, Clinical Frailty Scale; CKD, chronic kidney disease; FIM, Functional Independence Measure; GCS, Glasgow Coma Scale; GNRI, Geriatric Nutritional Risk Index; GOS, Glasgow Outcome Scale; GOSE, Glasgow Outcome Scale-Extended; HFRS, Hospital Frailty Risk Score; MCID, minimally clinically important difference; MDC, minimal detectable change; TBI, traumatic brain injury

Outcome category	Studies and key findings (effect estimates/p-values)
Functional recovery	OR 2.1, 95% CI 1.59-2.77, p < 0.001 - Frailty was linked to unfavorable long-term GOSE [10]. OR 9.9, 95% CI 2.1-46.3, p = 0.002 - Frailty was linked to unfavorable outcomes in moderate-to-severe TBI [8]. OR 0.645, 95% CI 0.595-0.699; OR 0.221, 95% CI 0.157–0.311, p < 0.001 - Intermediate and high frailty were linked to lower odds of achieving Barthel Index ≥95 at discharge [6]. OR 1.85, 95% CI 1.10-3.12 - Higher cognitive function indicated a greater likelihood of clinically meaningful motor improvement; OR 0.88, 95% CI 0.79-0.98 - Greater comorbidity burden suggested lower odds of improvement [25]. β = 0.03, 95% CI 0.002-0.06, p = 0.034 - Higher discharge mobility was linked to more healthy days at home [15]. p = 0.007 - CKD was linked to lower discharge GCS; p = 0.004 - CKD was linked to higher hospital-acquired infection rates. OR 1.30, 95% CI 1.06-1.60; OR 0.56, 95% CI 0.37-0.85; OR 1.47; OR 1.01 - Older age, lower admission GCS, elevated serum urea, and higher creatinine indicated factors related to poorer outcomes [28]. OR 0.77, 95% CI 0.71-0.84, p < 0.001 - Higher GNRI was linked to lower odds of incomplete six-month recovery; OR 0.047, 95% CI 0.020-0.109, p < 0.001 - GNRI ≥97.85 was linked to lower odds of incomplete recovery [4].
Discharge disposition and independence	aOR 2.63, 95% CI 1.02-7.14, p = 0.005 - High frailty was linked to unfavorable discharge disposition [31]. 24.4% developed post-injury ADL dependence and 17.5% required nursing home admission, with increasing age and comorbidity burden linked to poorer outcomes [10]. Community discharge increased from 67.8% to 71.6%, alongside improvements in self-care and mobility [32]. Patients discharged to nursing facilities had fewer healthy days at home (47.0% vs 77.7%) during the following year [25].
Hospital length of stay and complications	+1.95 days and +5.77 days - Intermediate and high HFRSs, respectively, were linked to progressively longer hospital stays [6]. 54.5% vs 7.4%, p = 0.004 - CKD was linked to higher rates of hospital-acquired infection; GCS 7.1 ± 5.9 vs 13.1 ± 2.6, p = 0.007 - CKD was linked to poorer neurological status at discharge [28]. Higher comorbidity and nutritional risk were also linked to greater clinical vulnerability [4,29].
Long-term and patient-reported outcomes	p = 0.026, p < 0.001, p = 0.033, and p = 0.038 - Injury severity was linked to GOSE, FIM cognition, FIM motor, and participation outcomes, respectively [16]. Lower educational attainment was linked to poorer global recovery, while severe acute headache was linked to poorer physical quality of life [3]. Frailty was also linked to lower life satisfaction and greater functional limitation [3].
Rehabilitation outcomes	84.1% achieved an MDC and 68.6% achieved an MCID in motor function [25]. OR 1.85, 95% CI 1.10-3.12 - Better baseline cognitive function indicated a greater likelihood of clinically meaningful motor improvement; OR 0.88, 95% CI 0.79-0.98 - Higher comorbidity burden suggested lower odds of achieving meaningful motor gains [25]. β = 0.03, 95% CI 0.002-0.06, p = 0.034 - Greater discharge mobility was linked to more days at home without healthcare utilization [30].

Mortality

Mortality was reported at several time points, including in-hospital, 30-day, 90-day, six-month, and longer-term follow-up. Frailty showed a strong association with mortality, particularly among patients with more severe TBI. In moderate-to-severe cases, those with CFS ≥4 had markedly higher six-month mortality than those with lower frailty scores (87% vs. 30%; OR 15.7, 95% CI 3.9-55.2, p < 0.001), and frailty remained independently associated after adjustment for clinical variables (OR 7.84, 95% CI 1.40-43.97, p = 0.019) [8]. Comorbidity burden also influenced mortality. Patients with American Society of Anesthesiologists (ASA) ≥3 had higher 90-day mortality (OR 3.44, 95% CI 1.10-13.41, p = 0.048), while age remained an independent predictor (OR 1.04 per year, 95% CI 1.00-1.09, p = 0.034) [26]. Among older adults with CSDH, mortality was higher than in matched population controls (HR 1.34, 95% CI 1.2-1.5, p < 0.0001). Frailty-related factors, including frequent falls (HR 1.3, 95% CI 1.0-1.7), inability to live independently (HR 1.4, 95% CI 1.1-1.8), inability to perform daily self-care (HR 1.5, 95% CI 1.1-1.9), and greater medication burden (HR 1.0, 95% CI 1.0-1.1), suggested an increased risk of mortality. Higher age (HR 1.05 per year) and CCI scores (HR 1.3 per point) similarly indicated a greater likelihood of mortality [27]. Overall, mortality was consistently associated with frailty, comorbidity burden, advanced age, and nutritional vulnerability, with the clearest quantitative effect seen in severe frailty among moderate-to-severe TBI cases.

Functional Recovery

Functional recovery was assessed using the Glasgow Outcome Scale (GOS), GOSE, Functional Independence Measure (FIM), and ADL-based measures. Frailty was consistently linked to poorer recovery. In mild TBI, frail patients had much lower rates of complete recovery than non-frail peers (24% vs. 72%), and frailty independently predicted unfavorable long-term GOSE (OR 2.1, 95% CI 1.59-2.77, p < 0.001) [10]. Among moderate-to-severe cases, unfavorable outcomes occurred in 92% of frail patients compared with 51% of non-frail patients (OR 9.9, 95% CI 2.1-46.3, p = 0.002) [8]. Higher frailty measured by the HFRS was also associated with poorer recovery. Compared with patients with low frailty, those with intermediate and high frailty had lower odds of achieving a Barthel Index ≥95 at discharge (OR 0.645, 95% CI 0.595-0.699 and OR 0.221, 95% CI 0.157-0.311, respectively; p < 0.001) and smaller improvements overall [6]. Baseline cognitive and functional status showed similar trends. Higher cognitive function increased the likelihood of clinically meaningful motor improvement during rehabilitation (OR 1.85, 95% CI 1.10-3.12), whereas greater comorbidity burden reduced it (OR 0.88, 95% CI 0.79-0.98) [25]. Higher mobility scores at discharge were associated with more healthy days at home during the following year (β = 0.03, 95% CI 0.002-0.06, p = 0.034) [15]. CKD appeared to be linked with poorer neurological recovery. Patients with CKD tended to have lower discharge GCS scores than those without CKD (7.1 ± 5.9 vs. 13.1 ± 2.6, p = 0.007) and showed higher rates of hospital-acquired infection (54.5% vs. 7.4%, p = 0.004). In multivariable analysis, older age (OR 1.30, 95% CI 1.06-1.60), lower admission GCS (OR 0.56, 95% CI 0.37-0.85), elevated serum urea (OR 1.47), and higher creatinine levels (OR 1.01) were identified as factors that may contribute to poorer outcomes [28].

Nutritional status provided an additional prognostic signal. Each unit increase in the Geriatric Nutritional Risk Index (GNRI) was associated with lower odds of incomplete six-month recovery (adjusted OR 0.77, 95% CI 0.71-0.84, p < 0.001), and patients with GNRI ≥97.85 had substantially lower odds of incomplete recovery (OR 0.047, 95% CI 0.020-0.109, p < 0.001) [3]. Together, these findings show that frailty and pre-injury functional and cognitive impairment were consistently associated with poorer recovery, while higher comorbidity and nutritional risk further reduced the likelihood of functional improvement.

Discharge Disposition and Independence

Premorbid functional status and frailty were also linked to post-injury independence. Among older adults with nonoperatively managed TBI, high frailty was associated with unfavorable discharge disposition in 53.8% compared with 25.7% of patients with low frailty (aOR 2.63, 95% CI 1.02-7.14, p = 0.005) [12]. In a larger cohort, 24.4% developed post-injury ADL dependence and 17.5% required nursing home admission, with increasing age and comorbidity burden predicting poorer outcomes [31]. Among patients admitted to inpatient rehabilitation, community discharge increased from 67.8% to 71.6% over the study period, accompanied by improvements in self-care and mobility [32]. However, patients discharged to nursing facilities had significantly fewer healthy days at home during the following year (47.0% vs. 77.7%) [25].

Hospital Length of Stay and Complications

Higher frailty was associated with greater healthcare utilization. Patients with intermediate and high HFRSs had progressively longer hospital stays compared with those with low frailty, with adjusted increases of 1.95 and 5.77 days, respectively [6]. Comorbid conditions also contributed to complications. Older TBI patients with CKD had markedly higher rates of hospital-acquired infection than matched patients without CKD (54.5% vs. 7.4%, p = 0.004) and poorer neurological recovery at discharge (mean GCS 7.1 vs. 13.1, p = 0.007) [28]. Higher comorbidity and nutritional risk were similarly associated with greater clinical vulnerability [4,29].

Long-Term and Patient‑Reported Outcomes

Long-term outcomes were less frequently reported. Among older adults followed for several years after TBI, greater injury severity was associated with poorer GOSE, FIM cognition, FIM motor, and participation outcomes [16]. Premorbid disability also predicted poorer social participation. In mild TBI, lower educational attainment was independently associated with poorer global recovery, while severe acute headache correlated with poorer physical quality of life [3]. Frailty was further linked to lower life satisfaction and greater functional limitation during long-term follow-up.

Rehabilitation Outcomes

Rehabilitation outcomes were primarily reported in studies involving older adults admitted to inpatient rehabilitation. Most patients showed measurable functional improvement, with 84.1% achieving a minimal detectable change (MDC) and 68.6% reaching an MCID in motor function [25]. However, the extent of improvement varied according to premorbid status. A higher comorbidity burden reduced the likelihood of achieving clinically meaningful motor gains, while better baseline cognitive function increased it [25]. Similarly, greater mobility at discharge was associated with more days spent at home without healthcare utilization during the following year [30].

Risk of Bias Assessment

Further evaluation was performed using the Joanna Briggs Institute critical appraisal tool [35]. Fourteen studies had high methodological quality, while six studies were rated as moderate quality. To allow comparison across studies, JBI scores were converted into percentages by dividing the number of “Yes” responses by the number of applicable criteria for each study. Because different study designs use different JBI checklists, the percentages were calculated using the relevant tool-specific denominators. The mean score across all studies was 82.2%, suggesting good methodological reporting quality. Most studies defined their populations clearly and measured exposures and outcomes using valid and reliable methods. Prospective studies had the highest quality ratings due to clearly defined participant recruitment and adequate follow-up. One limitation was incomplete reporting of follow-up procedures, insufficient data, or discussion of confounding. Studies by Sharma et al. and Mo et al. received moderate ratings due to potential selection bias, as mentioned earlier, or limited reporting of methodological procedures [17,28]. Overall, the included studies most consistently reported associations between frailty, baseline functional impairment, comorbidity burden, and poorer mortality or functional outcomes, as shown in Table 4.

**Table 4:** Methodological quality assessment of included studies using the JBI critical appraisal tools. JBI scores reflect the number of criteria met out of the total applicable items for each study design (maximum 11 for cohort studies; maximum 10 for case-control studies). Quality ratings were assigned as follows: High (≥9/11 or ≥8/10) and Moderate (7-8/11 or equivalent). JBI, Joanna Briggs Institute [35]

Study	Design	JBI score	Quality rating
Dams-O’Connor et al. (2016) [2], USA	Prospective cohort	8/11	Moderate
Horvat et al. (2025) [3], Serbia	Cohort	10/11	High
Zhu et al. (2023) [4], China	Cohort	7/11	High
Yamamoto et al. (2022) [6], Japan	Cohort	9/11	High
Zacchetti et al. (2023) [8], Italy	Prospective cohort	10/11	High
Hernández-Durán et al. (2022) [9], Germany	Cohort	9/11	High
Abdulle et al. (2018) [10], Netherlands	Prospective cohort	10/11	High
Kumar et al. (2025) [15], India	Cohort	8/11	Moderate
von Seth et al. (2026) [16], Sweden	Cohort follow-up	10/11	High
Sharma et al. (2021) [17], India	Cohort	9/11	Moderate
Evans et al. (2022) [25], USA	Cohort	9/11	High
Kiwanuka et al. (2025) [26], Sweden	Cohort	9/11	High
Blaauw et al. (2022) [27], Netherlands	Cohort	10/11	High
Mo et al. (2023) [28], China	Case-control	8/10	Moderate
Su et al. (2021) [29], Taiwan	Cohort	7/11	Moderate
Kumar et al. (2024) [30], USA	Cohort	9/11	High
Sastry et al. (2022) [31], USA	Cohort	9/11	High
Deutsch et al. (2024) [32], USA	Database cohort	9/11	High
Gavrila Laic et al. (2023) [33], Belgium	Cohort	8/11	Moderate
Visen et al. (2024) [34], India	Cohort	10/11	High

Discussion

The occurrence of TBI among older adults is noteworthy because of the high incidence of chronic medical conditions and disorders of premorbid functional status in this population. Among the 20 included studies, frailty and premorbid functional impairment were the most consistent predictors of adverse outcomes in older adults with TBI. The strength of these associations varied by injury severity and outcome measure. Higher frailty scores were associated with poorer ADL recovery, unfavorable discharge disposition, and longer hospitalization [6,9]. A greater comorbidity burden, particularly when measured with ASA, was linked to increased mortality and reduced functional improvement, although the strength of the association varied across individual diseases. Nutritional vulnerability assessed by GNRI was associated with increased mortality and incomplete functional recovery. Overall, the evidence indicates that chronological age alone does not adequately determine outcomes after geriatric TBI. Instead, premorbid frailty, functional status, comorbidity burden, and nutritional status provide crucial prognostic insight into recovery and survival in this population.

Mortality

Mortality among individuals with TBI could be either in-hospital or 90-day mortality. The ASA score has been shown to be a good determinant of comorbid status in patients with TBI. A score greater than 3 in a patient signified severe comorbidities. This was associated with increased 90-day mortality in geriatric TBI patients (8.2%) compared with non-TBI patients [36]. This is similar to a 7% 90-day mortality among TBI patients older than 80 years [37]. In addition, 35% of TBI individuals demonstrated in-hospital mortality compared with 26% of non-TBI individuals. Frailty was also linked to TBI mortality, as measured using the CFS. High-risk frail patients with CFS ≥4 were characterized by premorbid diseases. They demonstrated six-month mortality of 87%, which was higher than the 30% observed in non-frail patients (OR 15.7; 95% CI 3.9-55.2, p < 0.002). These findings revealed that pre-injury comorbid status could predict mortality among geriatric TBI patients.

In terms of preexisting comorbidities, there was a higher proportion of coronary artery disease (21.5% vs. 13.1%, p = 0.026) and end-stage renal disease (11.2% vs. 4.0%, p = 0.003) in the mortality group than in the survival group [29]. This demonstrated that premorbid conditions could play a role in mortality outcomes in this population.

Functional Recovery

Functional recovery was measured using the GOS, GOSE, and Barthel Index for ADL. Frailty showed a consistent link with the ability to perform ADL, functional decline, and reduced quality of life. The intermediate- and high-frailty-risk groups showed an undesirable Barthel Index score of ≥95 at discharge (intermediate: OR 0.645; 95% CI 0.595-0.699; high-frailty group: OR 0.221; 95% CI 0.157-0.311) and lower Barthel Index gains (intermediate: coefficient -4.868, 95% CI -5.599 to -3.773; high-frailty-risk group: coefficient -19.596, 95% CI -22.242 to -16.714) compared with the low-frailty group. These findings suggest that the frailty score may serve as a prognostic tool for functional outcomes. Consequently, it could be employed as a functional prognostic tool [6].

The GCS at presentation among older adults with low GNRI (<85) was lower than that among those with higher GNRI (median (IQR): 13 vs. 15, respectively; p < 0.001) at presentation to the emergency unit [29]. This indicated that the severity of injury among those with high nutritional risk was greater. Also, GOS at discharge among TBI patients was worse, which likely reflected the high ASA scores resulting from pre-injury chronic diseases. A GOS of 5 was attained by 1% of TBI patients compared with 39% of non-TBI individuals [36]. Frailty measured using the mFI-5 tool was associated with functional limitation among elderly frail and non-frail TBI patients (69.6% vs. 47.2%; p < 0.001) [36].

Discharge Disposition

Unfavorable discharge encompasses in-hospital death, nursing facility discharge, or discharge home with hospice. Evidence from a retrospective study conducted on 100 patients in 2022 showed that frailty was associated with undesirable discharge disposition. It revealed that patients with high frailty were more predisposed to an adverse discharge disposition (53.8% vs. 25.7%, p = 0.007) than those with low frailty. Also, high frailty (aOR 2.63, 95% CI 1.02-7.14, p < 0.005) was significantly associated with poor discharge outcomes [31]. The GNRI is a metric for measuring nutritional status in older patients. Multiple investigations have shown that it can be employed as a tool to assess recovery in TBI. In a study conducted in 2023 involving 228 patients aged 65 years and older, the GOSE was used as the recovery tool. It showed that higher GNRI was associated with a lower risk of incomplete recovery at six months (OR 0.770, 95% CI 0.709-0.837, p < 0.001) [4] compared with low GNRI (<85) [29].

*Duration of Hospital Stay and Complication*s

The HFRS has been validated as a tool for identifying patients at risk of unfavorable outcomes and frailty following TBI. High-risk scores were defined as HFRS >15, intermediate risk as 5-15, and low risk as <5. In a retrospective cohort study conducted in 2022 on the effect of frailty risk on negative outcomes following elderly TBI in 18,065 hospitalized patients, higher and intermediate HFRS risk groups (high: coefficient 5.770, 95% CI 3.160-8.379; intermediate: coefficient 1.952, 95% CI 1.117-2.786) had prolonged hospital stays compared with the low-frailty group [6]. Similarly, an observational study conducted in 2018 reported that among hospitalized acute-care patients older than 75 years, those with HFRS had approximately sixfold longer hospital stays than those with LFRS [38]. These outcomes confirmed that a high frailty score is associated with prolonged hospital stay. Another study demonstrated that patients with GNRI <85 had a prolonged hospital stay (25.2 days vs. 18.6 days, p = 0.004) and higher mortality rates compared with patients with higher GNRI >103 [29].

Comorbidities and Complications

CKD in elderly TBI patients was associated with lower GCS scores at discharge and hospital-acquired infection. In a study conducted in 2023, the CKD TBI group had a significantly lower GCS score compared with the non-CKD group (13.1 ± 2.6). Additionally, CKD TBI patients had more hospital-acquired infections (7.4%, p < 0.01) compared with non-CKD patients [28]. This showed that elderly TBI patients with underlying renal dysfunction had poorer outcomes and might be at risk of infection compared with those without CKD. In a study that evaluated hospital stay in patients with pre-injury diabetes mellitus, a higher proportion of TBI patients with underlying diabetes had a prolonged hospital stay: 26.7% stayed for >7 days (OR 1.13, 95% CI 1.01-1.26) and 14% stayed for >14 days (OR 1.09, 95% CI 0.95-1.25), compared with 19.9% and 10.0%, respectively, among those without DM [39].

Anticoagulant use is quite common among the geriatric population because of its use for several comorbidities, such as atrial fibrillation. A prospective study involving 220 patients ≥60 years demonstrated that anticoagulant use was correlated with poor outcomes. In elderly TBI patients on anticoagulants, there was a strong association with unfavorable outcomes (4% vs. 0%, p < 0.005). In addition, pre-injury coagulation abnormality was associated with unfavorable TBI outcomes and worsened TBI findings on repeat imaging (OR 3.22, 95% CI 1.72-6.02, p < 0.001) compared with those without coagulation abnormalities [34]. It was also found to be significantly predictive, as it was associated with an increased risk of hemorrhage in TBI patients [34]. Therefore, prompt reversal at presentation is associated with better outcomes.

Rehabilitation following geriatric TBI is strongly influenced by baseline comorbidities and duration of hospital stay. Rehabilitation outcomes were measured using MDC and MCID among elderly TBI patients after rehabilitation. The study showed that 84% attained the MDC threshold and 68% attained the MCID threshold following inpatient rehabilitation facility admission [25]. This further underscores the essential role rehabilitation plays following TBI in elderly patients. However, comorbidity burden and baseline functional status should be used as tools for care planning. Frailty and comorbidity indices may better predict TBI outcomes in older adults than chronological age. This is because chronic medical conditions are more common at this age and affect outcomes such as mortality, hospitalization, and recovery. A scale that encompasses both frailty and comorbidities may serve as a useful prognostic tool for identifying outcomes in each geriatric TBI patient, unlike age alone, which can be a rigid predictor of TBI outcomes. Indices such as the CFS, HFRS, and eTBI are validated tools that predict adverse outcomes and mortality, measured by the GOSE, and account for comorbid conditions; these have been shown to outperform age in geriatric patients [40].

Frailty is not just an effect of aging but comprises factors relating to comorbidities. Observational studies have consistently shown that frailty indices are better predictors of outcomes than chronological age. In an observational cohort study involving elderly patients older than 60 years with mild TBI, the study found that 72% of non-frail elderly patients fully recovered compared with 24% of frail patients (p < 0.01) [10]. Also, at 1-3 years post-injury, frail elderly patients reported lower mean life satisfaction scores (6.7 vs. 7.9, p < 0.01). This further emphasizes the consideration of frailty and comorbidity indices instead of chronological age. Similarly, another study revealed that factors such as frailty and comorbidities should be considered in the prognosis and management of patients and that age alone does not predict poor outcomes [41].

Prognostic Models 

The two major TBI prognostic models are the International Mission for Prognosis and Analysis of Clinical Trials in TBI (IMPACT) and the Corticosteroid Randomization After Significant Head Injury (CRASH) [42,43]. These models predict patient outcomes after TBI, but both CRASH and IMPACT underestimate and overestimate outcomes in geriatric patients, respectively. They also do not measure baseline frailty and chronic disorders. The burden of chronic illnesses such as cardiovascular disease, renal disease, and diabetes is particularly higher in the elderly population, with an effect on TBI outcomes. Therefore, there is a vital need for a tool that assesses these factors for prognosis in geriatric TBI patients [14]. The Modified Frailty Index and CCI are assessment tools that measure frailty with a focus on comorbidities but are limited by their lack of nutritional and cognitive assessment [44,45]. They are validated tools used primarily to assess adverse surgical outcomes.

In a case-control study of elderly TBI patients in 2024 that evaluated the predictive power of eTBI, a tool predictive of functional outcome in geriatric TBI with consideration of comorbidities, the score was used to predict one-month outcomes in geriatric patients. The score stratified patients into low-, medium-, and high-risk groups. At 30 days, 85% of the low-risk group had some level of improvement, while 100% of the high-risk group became vegetative. The eTBI had an accuracy of 88% and specificity of 98%. Premorbid functional status, cognition, nutritional status, and preexisting comorbidities are important determinants of recovery and survival in geriatric TBI; therefore, there is a need for these factors to be incorporated into the assessment and management of geriatric TBI, as they better predict outcomes and provide prognostic insight in elderly TBI patients [40].

Strength and Limitations

This review corroborated evidence from a broad range of recent observational non-randomized studies. The inclusion of elderly patient populations with variable comorbidities, the effects of frailty indices, and the different impacts these factors have on the geriatric population emphasized the consistent necessity of a standardized metric scale that considers pre-injury comorbidities. Differences in frailty indices used by several studies caused a limitation in result comparability. Some studies used CFS and HFRS, while others used FRAIL scores. In addition, comorbidity reporting across several studies was inconsistent; publication and language biases may have excluded some relevant studies, and numerous studies also lacked geriatric relevance. There was also variability in adverse outcome reporting, which could have underestimated the actual frequency of these occurrences. In spite of these limitations, the collective evidence reinforces the clinical pertinence of the impact of pre-injury comorbidities in elderly TBI patients. Consequently, the observed findings across several non-randomized studies further underscore the necessity of a standardized metric scale tailored to each geriatric TBI patient. One that includes comorbidity and premorbid function components could serve as a prognostic tool to predict patient outcomes.

## Conclusions

This systematic review indicates that premorbid frailty, functional dependence, comorbidity burden, and nutritional vulnerability are consistently associated with adverse outcomes following TBI in older adults. Frailty was associated with higher mortality, poorer functional recovery, unfavorable discharge disposition, and longer hospital stay across different TBI populations. Greater comorbidity burden and specific conditions, including CKD, were also associated with poorer neurological recovery and higher complication rates, while better premorbid cognitive and functional status was associated with greater rehabilitation gains. The findings suggest that chronological age alone does not adequately capture the heterogeneity of outcomes following geriatric TBI. Assessment of frailty, premorbid functional and cognitive status, comorbidity burden, and nutritional status may provide additional prognostic information and should be considered during clinical assessment, rehabilitation planning, and discussions regarding expected recovery. However, differences in age definitions, TBI severity, frailty instruments, comorbidity measures, and outcome definitions limit direct comparison across studies.

Future prospective research should develop and validate geriatric-specific TBI prognostic models incorporating premorbid function, frailty, comorbidity burden, cognition, and nutritional status alongside established measures of injury severity. Such models should be evaluated for their ability to improve risk stratification and guide individualized rehabilitation and post-acute care.
